# Influenza Virus Induces Cholesterol-Enriched Endocytic Recycling Compartments for Budozone Formation via Cell Cycle-Independent Centrosome Maturation

**DOI:** 10.1371/journal.ppat.1005284

**Published:** 2015-11-17

**Authors:** Atsushi Kawaguchi, Mikako Hirohama, Yoshimi Harada, Suguru Osari, Kyosuke Nagata

**Affiliations:** 1 Department of Infection Biology, Faculty of Medicine, University of Tsukuba, Tennodai, Tsukuba, Japan; 2 Graduate School of Comprehensive Human Sciences, University of Tsukuba, Tennodai, Tsukuba, Japan; Icahn School of Medicine at Mount Sinai, UNITED STATES

## Abstract

Influenza virus particles are assembled at the plasma membrane in concert with incorporation of the virus genome, but the details of its spatio-temporal regulation are not understood. Here we showed that influenza virus infection induces the assembly of pericentrosomal endocytic recycling compartment (ERC) through the activation of Rab11a GTPase and cell cycle-independent maturation of centrosome by YB-1, a multifunctional protein that is involved in mitotic division, RNA metabolism and tumorigenesis. YB-1 is recruited to the centrosome in infected cells and is required for anchoring microtubules to the centrosome. We also found that viral infection accumulates cholesterol in ERC and is dependent on YB-1. Depletion of YB-1 shows reduced cholesterol-enriched ERC and prevented budozone formation at the plasma membrane. These results suggest that cholesterol in recycling endosomes, which are emanated from ERC, may trigger the virus assembly concomitantly with the packaging of the virus genome. We propose that the virus genome is transported to the plasma membrane by cholesterol-enriched recycling endosomes through cell cycle-independent activation of the centrosome by YB-1.

## Author Summary

Influenza virus particles are assembled at the plasma membrane in concert with incorporation of the virus genome, but the details of its spatiotemporal regulation are unknown. We found that the virus genome is transported to the plasma membrane using cholesterol-enriched recycling endosomes through cell cycle-independent activation of the centrosome by recruiting YB-1, which is a mitotic centrosomal protein. We also revealed that the cholesterol-enriched endosomes are important for clustering of viral structural proteins at lipid rafts to assemble the virus particles. These results suggest that local accumulation of cholesterol, via fusion of endosomes to the plasma membrane, is one of the triggers for the virus assembly concomitantly with arrival of the virus genome beneath the plasma membrane.

## Introduction

Endocytic transport pathways are important to arrange the plasma membrane components for diversified cellular processes at the plasma membrane including virus budding. Endocytosed proteins are first delivered to the early/sorting endosomes, from where proteins are either recycled back to the plasma membrane or transported to late endosomes and lysosomes. Rab small GTPase family members show distinct intracellular localization and function as molecular switches to regulate vesicle carrier formation and fusion with target membranes. Rab11a-positive recycling endosomes are crucial for recycling and delivery of plasma membrane components to the cell surface [1–3]. The Rab11a-positive transport vesicles emerge from specific organelles called endocytic recycling compartments (ERC). ERCs constitute a collection of tubular organelles that are close to the nucleus and associated with the microtubule organizing centre (MTOC). However, the functional significance of ERCs is not fully understood.

MTOC is a highly dynamic structure that achieves precise control of the microtubule array for the spatial and temporal regulation of several fundamental processes. Microtubule dynamics is controlled through continuous switching between phases of growth and shrinkage, as well as the level and timing of nucleation from the centrosome, which is the major MTOC in animal cells. The centrosome is composed of a pair of centrioles surrounded by pericentriolar material (PCM), a matrix of more than a hundred different proteins. PCM proteins are organized radially around the centriole in a toroid-like arrangement [4–7] and PCM serves as a platform for microtubule nucleation. During mitosis, in a process known as centrosome maturation, PCM increases in size to promote the microtubule nucleation for mitotic spindle formation [8,9].

The influenza viral genome forms a viral ribonucleoprotein complex (vRNP) with viral RNA polymerases and nucleoprotein (NP). After viral genome replication in the nucleus, the progeny vRNP is nuclear-exported and then accumulates around the centrosome [10]. vRNP is then transported to the budding site beneath the cell surface along microtubules through Rab11a-dependent recycling endosomes [11–13]. Recently, Y-box binding protein-1 (YB-1) was reported to function as a porter to facilitate vRNP accumulation at the centrosome [14]. YB-1 is a major component of cellular mRNA ribonucleoprotein complexes and it regulates mRNA translation and degradation [15]. It is also reported that YB-1 accumulates in the centrosome during G2/M phases [16] and is required for the centrosome maturation [17].

Cholesterol is a major constituent of the plasma membrane in eukaryotic cells. It regulates the physical state of the plasma membrane and is involved in the formation of membrane microdomains, called lipid rafts. Lipid rafts are defined as small (10–200 nm), heterogeneous, highly dynamic, sterol- and sphingolipid-enriched domains that compartmentalize cellular processes [18]. Small rafts can sometimes coalesce to form larger platforms through protein-protein, protein-lipid, and lipid-lipid interactions. Three viral membrane proteins, HA, NA, and M2, are embedded in the influenza virus envelope. M1 covers the inner viral membrane leaflet and binds to the cytoplasmic tails of HA and NA [19]. The assembly and budding of viral particles are coupled with the formation of functionalized raft domains, called budozone [20]. In the budozone, HA, possibly together with NA, is enriched by clustering several small rafts [21,22]. M2 possesses cholesterol-binding motifs [23,24], but a relatively short transmembrane domain of M2 prevents complete immersion of the protein in the more ordered raft domains. Thus, M2 is thought to localize to the edge of the budozone to mediate the pinching off of virus particles from the plasma membrane [25]. Finally, vRNP is recruited to the budozone through the interaction of vRNP with M1 to initiate budding and release of virus particles.

Here we showed that influenza virus infection induces the assembly of pericentrosomal ERCs through the activation of Rab11a and microtubule dynamics. Using three-dimensional structured illumination microscopy (3D-SIM), we found that YB-1 forms a toroid-like structure with a beads-on-a-string distribution pattern around the centriole. Knockdown (KD) analyses indicated that influenza virus stimulates the spontaneous centrosome maturation in interphase by recruiting YB-1 to anchor newly synthesized microtubules onto the centrosome. We also found that cholesterol accumulates in the pericentrosomal ERC with vRNP in an YB-1-dependent manner. Disruption of the cholesterol-enriched ERC formation by YB-1 KD results in defective viral budozone formation at the plasma membrane. Collectively, these results suggest that the recycling endosomes containing cholesterol and vRNP emanate from ERC, and cholesterol in recycling endosomes is a trigger for the viral budozone formation concomitantly with vRNP trafficking to the plasma membrane.

## Results

### Influenza virus infection stimulates the pericentrosomal ERC formation

**Fig 1:**
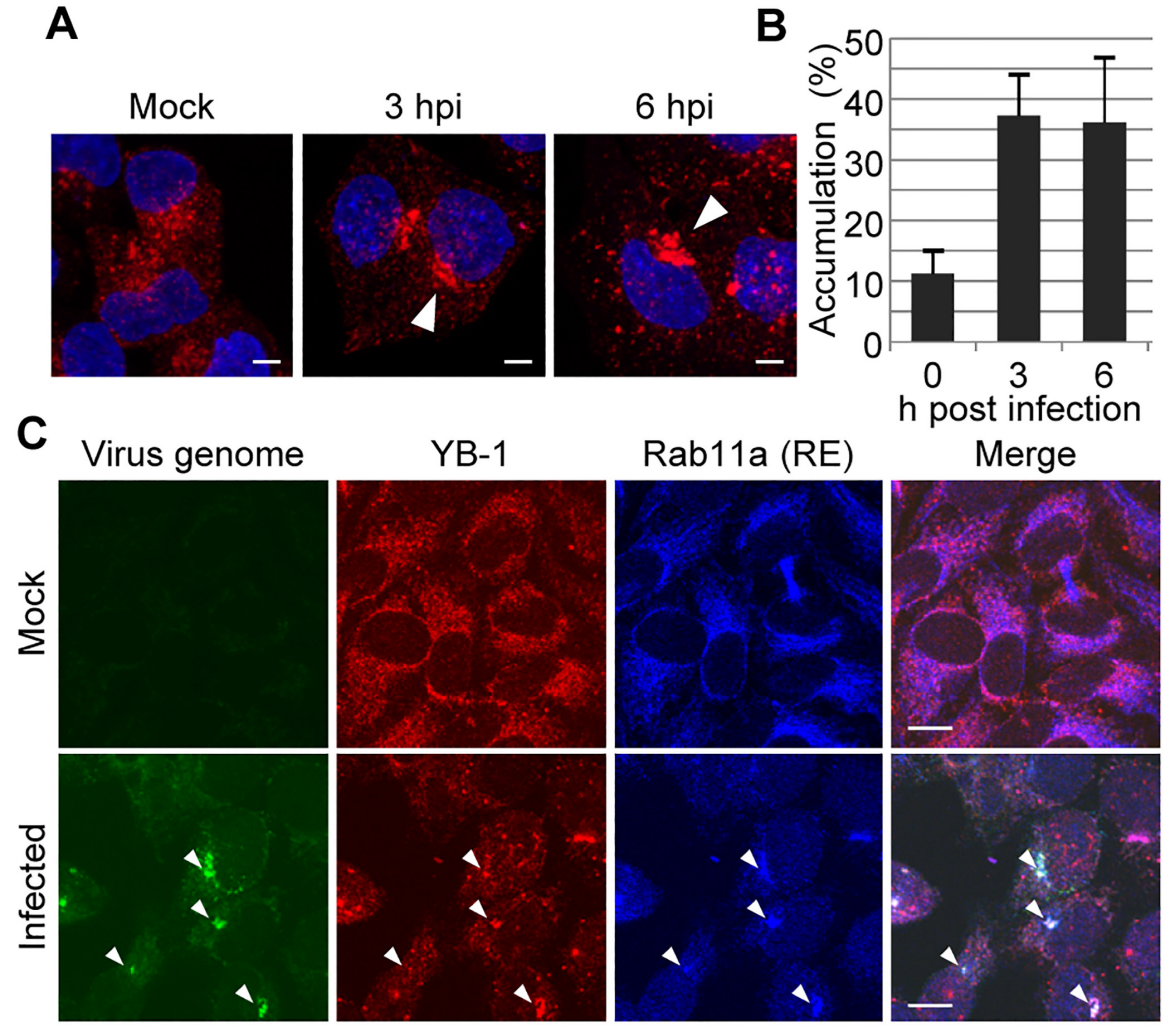
Formation of pericentrosomal ERC with the virus genome and YB-1 in infected cells. (A and B) Transferrin uptake. HeLa cells were pulse-labeled with 100 μg/ml of transferrin conjugated with Alexa 568 (red; arrowhead) for 30 min at 37°C at 0, 3, and 6 h post infection, respectively. After washing with medium, cells were further incubated for 30 min at 37°C. Nuclei were counter-stained with DAPI (blue). The average number of cells showing accumulation of transferrin larger than 1 μm was obtained from three independent experiments (panel B; *n* = 100). (C) Intracellular localization of the virus genome, YB-1, and Rab11a. At 6 h post infection, uninfected (upper panels) and infected HeLa cells (lower panels) were subjected to indirect immunofluorescence assays with anti-YB-1 (red) and anti-Rab11a (blue) antibodies, followed by FISH assays using an RNA probe complementary to the virus genome (green). The representative results from three independent experiments are shown. Scale bar, 10 μm.

Transferrin is a typical marker to monitor the organization of active recycling endosomes during endocytosis and its return to the cell surface. To examine the dynamics of the recycling pathway in influenza virus-infected cells, cells were pulse-labeled for 30 min with transferrin Alexa fluor 568, followed by a chase for 30 min without fluorescent transferrin. At 3 h post infection, transferrin-positive recycling endosomes were accumulated in ERC at a juxta-nuclear region, possibly near the centrosome (Fig 1A and 1B, white arrowheads). Transferrin recycling proceeds with a t_1/2_ of approximately 20 min [26], therefore the transferrin uptake should correspond to a steady-state distribution of the labeled ligand (Fig 1A). We next performed an indirect immunofluorescence assay using anti-Rab11a antibody and FISH assay using a probe that hybridizes with the segment 1 virus genome (Fig 1C, arrowheads). As is the case for transferrin, Rab11a was also present in the juxta-nuclear region and colocalized with the virus genome in approximately 40% of infected cells at 6 h post infection (*P*<0.001), suggesting that the virus genome is recruited to the pericentrosomal ERC after nuclear-export, as previously reported [10–14].

**Fig 2:**
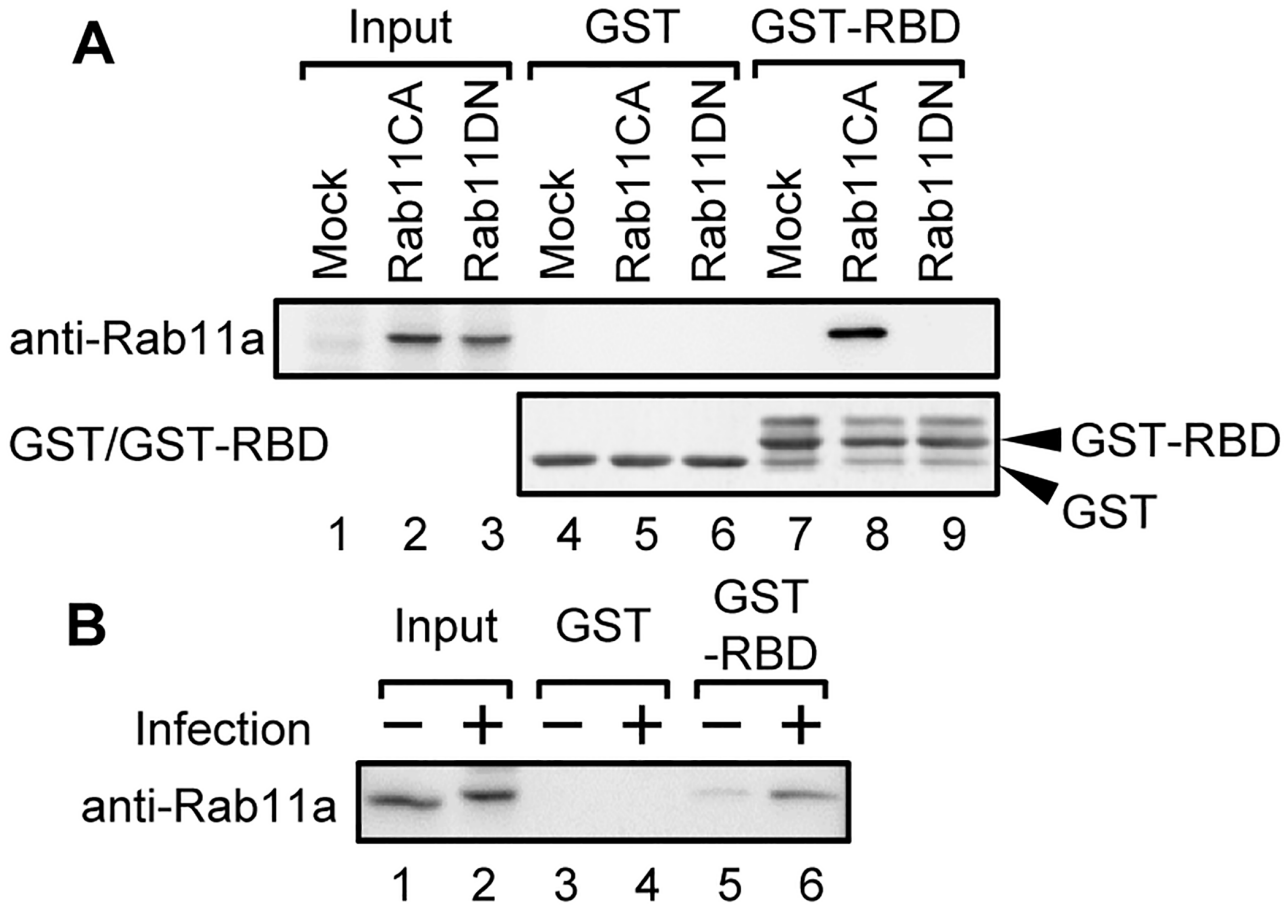
Activation of Rab11a GTPase in infected cells. (A) HeLa cells were transfected with a plasmid expressing either GFP (lanes 1, 4, and 7), GFP-fused Rab11a constitutive active mutant (CA; lanes 2, 5, and 8), or GFP-fused Rab11a dominant negative mutant (DN; lanes 3, 6, and 9). At 24 h post transfection, cell lysates were prepared and subjected to GST pull-down assays with either GST (lanes 4–6) or GST-RBD (lanes 7–9). (B) At 8 h post infection (MOI = 10), mock-infected (lanes 1, 3, and 5) and infected HeLa cells (lanes 2, 4, and 6) were subjected to GST pull-down assays with either GST (lanes 3 and 4) or GST-RBD (lanes 5 and 6). Co-purified proteins were detected by western blotting assays with anti-Rab11a antibody. GST and GST-RBD were detected by CBB staining.

It has been shown that active Rab11a shows a marked accumulation of ERC at the centrosome [27]. To evaluate whether Rab11a is activated by influenza virus infection, we purified active Rab11a (Rab11-GTP) by GST pull-down assays using Rab11-binding domain of Rab11-FIP2. Rab11-FIP2 acts as an effector molecule for Rab11-GTP through a highly conserved Rab11-binding domain (RBD) among Rab11-FIP family proteins [28]. Therefore, we can purify Rab11-GTP (constitutive active mutant Q70L, lane 8), but not the GDP form (dominant negative mutant S25N, lane 9), using GST-fused 41 amino acid peptide derived from RBD of Rab11-FIP2 (GST-RBD) (Fig 2A). Next, we performed GST pull-down assays with lysates prepared from infected cells using GST-RBD at 8 h post infection (at which the virus genome is actively transported) and the co-purified Rab11a was analyzed by western blotting with anti-Rab11a antibody (Fig 2B). The amount of Rab11a co-purified with GST-RBD from infected lysates was 4.5 ± 0.6 times more than that from mock-treated lysates (Fig 2B; representative results from three independent experiments are shown), suggesting that a guanine nucleotide exchange factor (GEF) for Rab11a may be activated in response to infection.

### Centrosome maturation by YB-1 is required for ERC formation

By interacting with a number of Rab11-FIPs, Rab11a associates with distinct motor proteins, enabling bidirectional transport along microtubules. Thus, recycling endosomes closely associate with microtubules, and their intracellular transport is fully dependent on the microtubule dynamics, which undergo cycles of nucleation, growing, and shrinking. The precise spatial and temporal regulation of the cycles is essential for the numerous cellular functions in which microtubules are involved.

**Fig 3:**
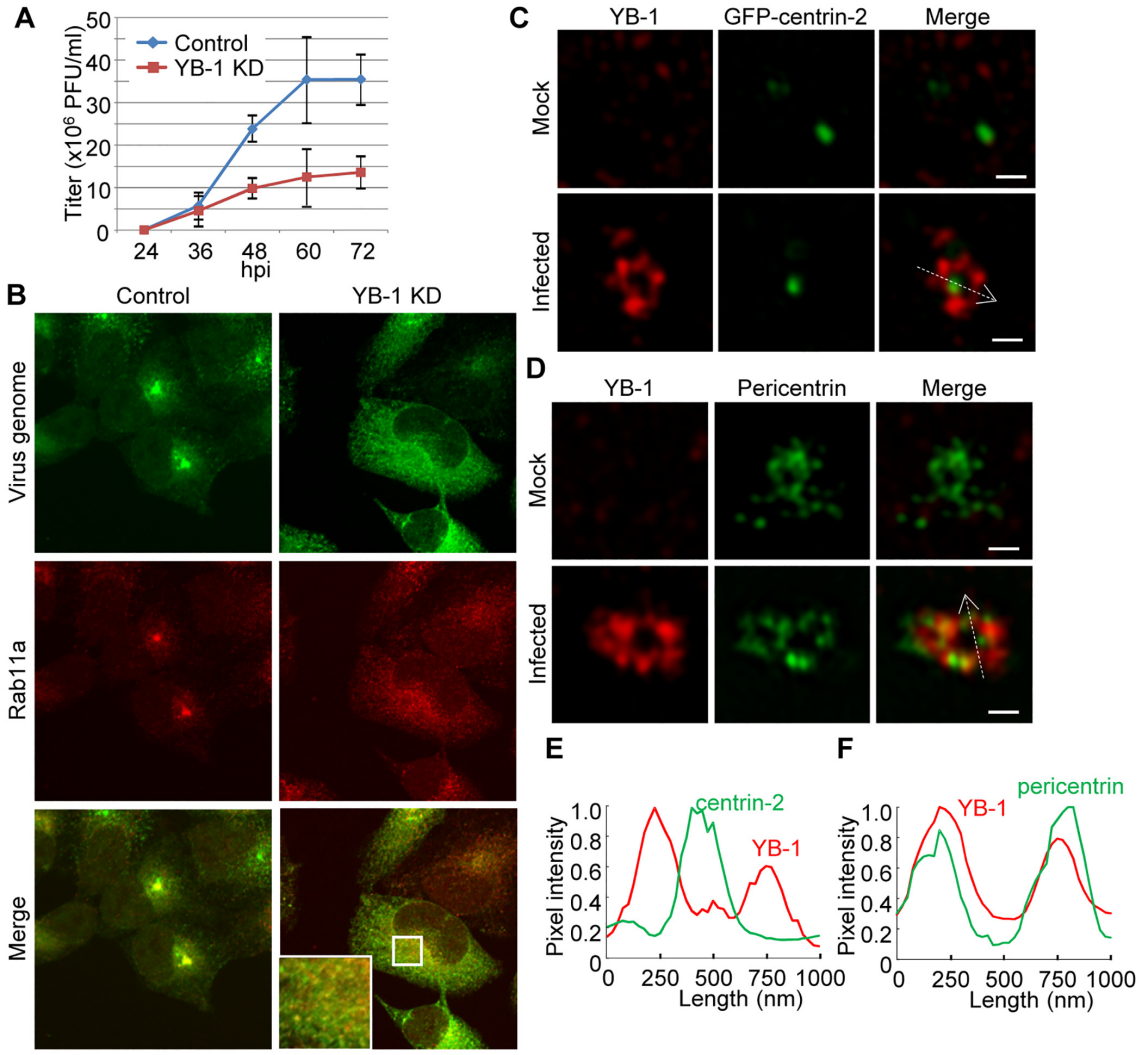
YB-1 is required for pericentrosomal ERC formation. (A) Production of infectious virions. HeLa cells were transfected with non-targeting or YB-1 siRNA. At 48 h post transfection of siRNA, control and YB-1 KD cells were infected with influenza virus at MOI of 0.01. The culture supernatants collected at 24, 36, 48, 60, and 72 h post infection were subjected to plaque assays to examine the production of infectious virions. The average titers and standard deviations determined from three independent experiments are shown. (B) Intracellular localization of the virus genome and Rab11a in YB-1 KD cells. At 48 h post transfection of siRNA, control and YB-1 KD HeLa cells were infected with influenza virus at MOI of 10. At 8 h post infection, immunofluorescence assays were carried out with anti-Rab11a antibody (red), followed by FISH assays using an RNA probe complementary to segment 1 virus genome. Scale bar, 5 μm. (C and E) Centrosomal localization of YB-1 and GFP-centrin-2. At 8 h post infection (MOI = 10), uninfected and infected HeLa cells constitutively expressing GFP-centrin-2 were subjected to indirect immunofluorescence assays with rabbit anti-YB-1 antibody (red). In panel E, quantitative determination of the each signal (centrin-2, green; YB-1, red) was performed along the white dashed arrows shown inside the panel E, bottom panel. Y-axis indicates the normalized pixel intensity. (D and F) Centrosomal localization of YB-1 and pericentrin. At 8 h post infection (MOI = 10), uninfected and infected cells were subjected to indirect immunofluorescence assays with mouse anti-pericentrin (green) and rabbit anti-YB-1 (red) antibodies. In panel F, quantitative determination of the each signal (pericentrin, green; YB-1, red) was performed along the white dashed arrows shown within the panel E, bottom panel. Y-axis indicates the normalized pixel intensity. In panel C and D, all images were acquired with a super-resolution microscopy (3D-SIM; Carl Zeiss). Scale bar, 500 nm.

Previously, we reported that YB-1 accumulates in the centrosome with vRNP during interphase [as shown in Fig 1C, and [14]]. At 48 h post transfection of YB-1 siRNA, the expression level of YB-1 in KD cells decreased to 25% of that in control cells (S1 Fig). The virus titer in YB-1 KD cells decreased to approximately 30% of that in control cells (Fig 3A). We also found that Rab11a does not accumulate in the centrosome of infected YB-1 KD cells (Fig 3B), suggesting that YB-1 is required for pericentrosomal ERC formation. Note that YB-1 is responsible for centrosome maturation in order to establish the polarity-dependent dynamic instability in the mitotic phase [17]. Thus, we hypothesized that YB-1 may stimulate pericentrosomal ERC formation through spontaneous centrosome maturation in infected interphase cells as it does in the mitotic phase. To test this hypothesis, we examined the centrosomal localization of YB-1 using 3D-SIM super-resolution microscopy (Fig 3C, 3D, 3E and 3F). Note that only centrosomes showing a cross-sectional view of PCM during interphase were selected for this analysis. YB-1 formed a toroidal structure with a beads-on-a-string distribution pattern around GFP-centrin-2, a marker protein of the centriole (Fig 3C and 3E). The mean diameter of the YB-1 toroid at the peak intensity (545 ± 48 nm; *n* = 8) was similar to that of pericentrin toroid (a marker for PCM; 581 ± 42 nm; *n* = 8), suggesting that YB-1 localizes in PCM (Fig 3D and 3F). However, YB-1 did not co-localize with pericentrin (Fig 3E). It has been proposed that pericentrin exists as elongated fibrils that extend radially from the centriole [5,6]. The spatial domains separated by pericentrin are filled with a number of PCM proteins required for microtubule nucleation and anchoring, suggesting that YB-1 also regulates the microtubule nucleation and/or anchoring at PCM in response to infection at interphases.

**Fig 4:**
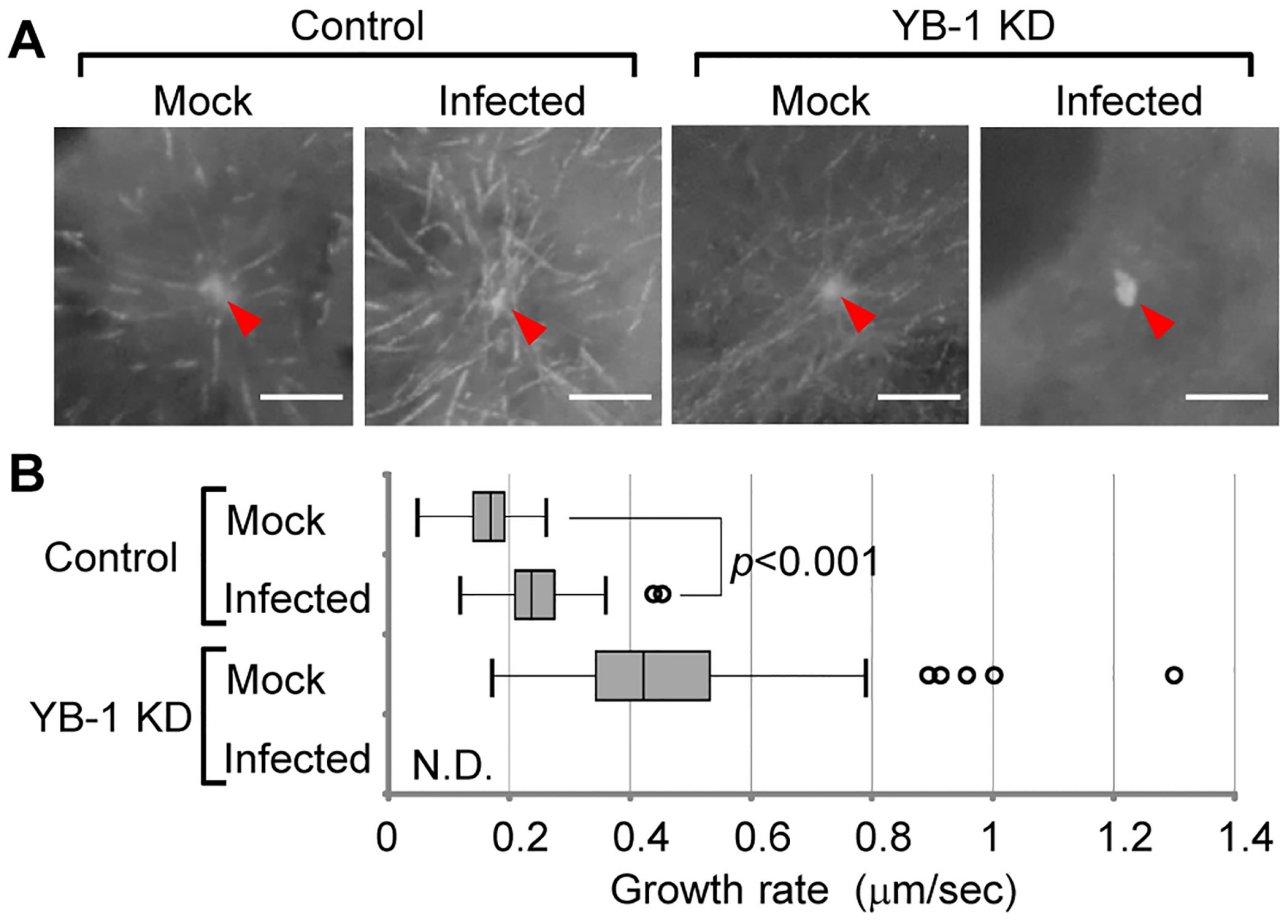
Microtubule nucleation from the centrosome visualized by EB1-GFP. (A) Live-cell imaging of EB1-GFP nucleated from the centrosome. After 48 h post treatment of either non-targeting or YB-1 siRNA, HeLa-EB1-GFP cells were infected with influenza virus. At 8 h post infection (MOI = 10), the cells were subjected to the live-cell imaging using confocal microscopy. Images were acquired at 1.56-sec intervals for 1 min (see also S1, S2, S3 and S4 Videos). The stack examples of each time-lapse image are shown (panel A). Red arrowheads indicate the position of the centrosome. Scale bar, 5 μm. (B) The quantitative results of growth rate distribution of EB1-GFP particles (over 80 particles obtained from more than ten cells) were determined from three independent experiments. The level of significance was determined by Student’s *t* test. N.D., not detectable.

Next, we observed the dynamics of microtubule nucleation to examine the centrosome function in infected cells using EB1-GFP [8], which interacts specifically with growing microtubule ends (Fig 4 and S1, S2, S3 and S4 Videos). The time series of EB1-GFP were acquired at 1.56-sec intervals for 1 min. In image sequences, EB1-GFP comets continually emerged from the centrosome. In the control, the mean growth rate of nucleated microtubules in the infected cells was increased compared to that of the uninfected mock cells (Fig 4B, *P*<0.001). In contrast, EB1-GFP in infected cells treated with YB-1 siRNA mostly did not move in a straight line, but rather in a Brownian motion (Fig 4A and S4 Video). Because growing microtubule ends decorated with EB1-GFP accumulated primarily in the centrosome of infected YB-1 KD cells (Fig 4A, arrow head), it is likely that the microtubules nucleated from the centrosome even in infected YB-1 KD cells. Therefore, it is possible that the newly synthesized microtubules are released from the centrosome in infected YB-1 KD cells. Further, although most microtubules were still elongated radially from the centrosome (Fig 4A), some of the EB1-GFP signals showed a faster migration rate in uninfected YB-1 KD cells (Fig 4A and 4B). It has been reported that short microtubules released from the centrosome migrate faster than the centrosomal microtubules [29], therefore YB-1 appears to be required, at least in part, for anchoring microtubules to the centrosome in uninfected interphase cells.

**Fig 5:**
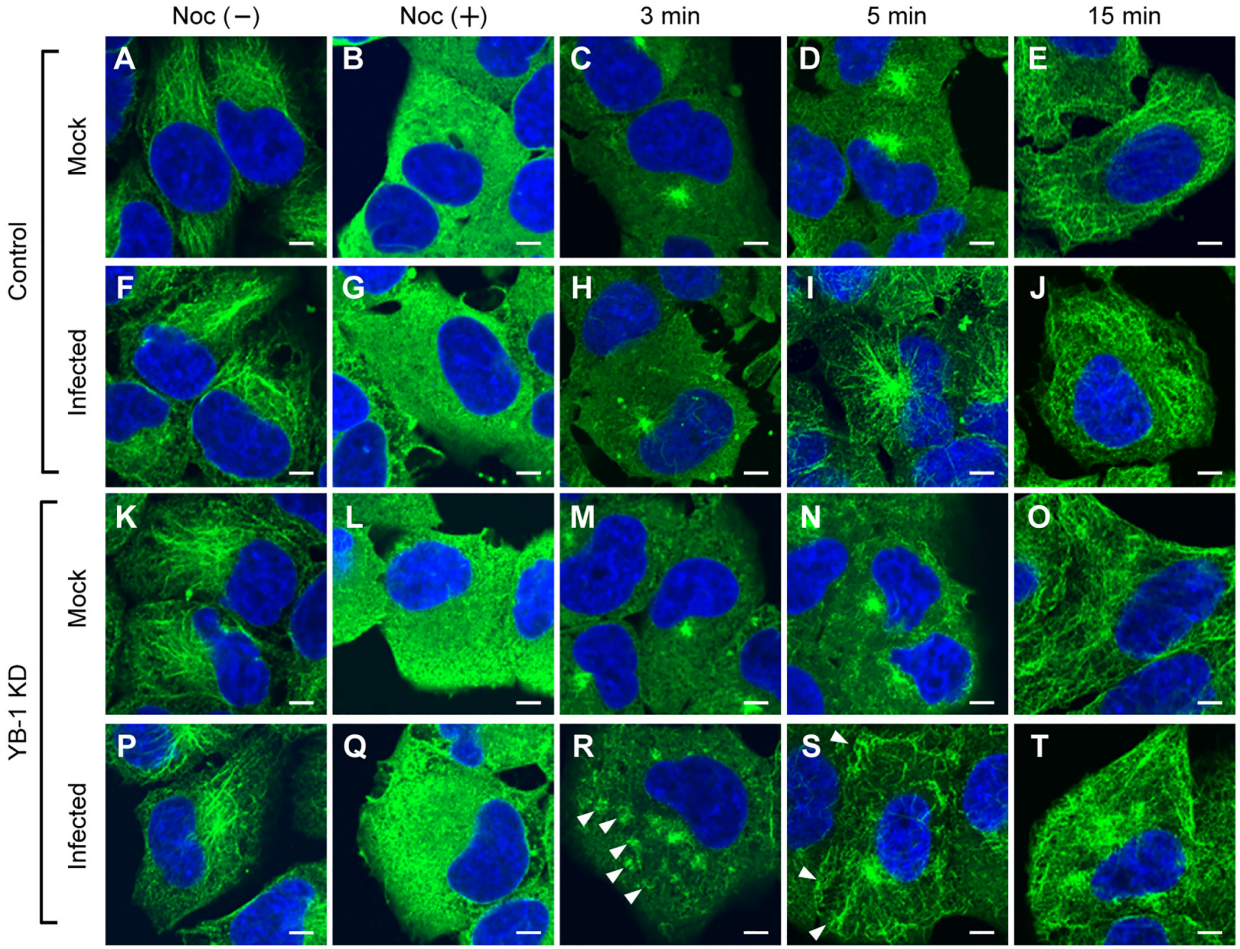
Microtubule nucleation after releasing from nocodazole treatment. At 48 h post transfection with either non-targeting (panel A-J) or YB-1 siRNA (panel K-T), HeLa cells were infected with influenza virus at MOI of 10. At 8 h post infection, cells were treated for 1 h without (Noc(-)) or with 1 μg/ml of nocodazole (Noc(+)). At 3 (panel C, H, M, and R), 5 (panel D, I, N, and S), and 15 min (panel E, J, O, and T) post releasing from nocodazole treatment, cells were fixed and subjected to indirect immunofluorescence assays with mouse anti-α-tubulin antibody. Arrowheads indicate the microtubules sporadically localized at the peripheral cytoplasm (panel R and S). Scale bar, 5 m.

To address whether YB-1 is involved in the anchoring of microtubules to the centrosome in response to infection, we carried out microtubule regrowth assays using nocodazole, a potent inhibitor of microtubule polymerization (Fig 5). After nocodazole treatment for 1 h, microtubules were depolymerized, and α-tubulin was dispersed throughout the cytoplasm (Fig 5B, 5G, 5L and 5Q). After washing out the drug, cells were incubated at 37°C to allow the regrowth of the microtubules for 3, 5, and 15 min. As expected, the nucleation of microtubules from the centrosome was stimulated by infection in control cells at 5 min post release (Fig 5I). In contrast, noncentrosomal microtubules were sporadically found at peripheral regions of the cytoplasm in infected YB-1 KD cells (Fig 5R and 5S, arrowheads). These results suggest that YB-1 is required for anchoring newly polymerized microtubules to PCM when the microtubule nucleation is stimulated by infection.

### Pericentrosomal ERC is important for enrichment of vRNP and cholesterol as cargo

**Fig 6:**
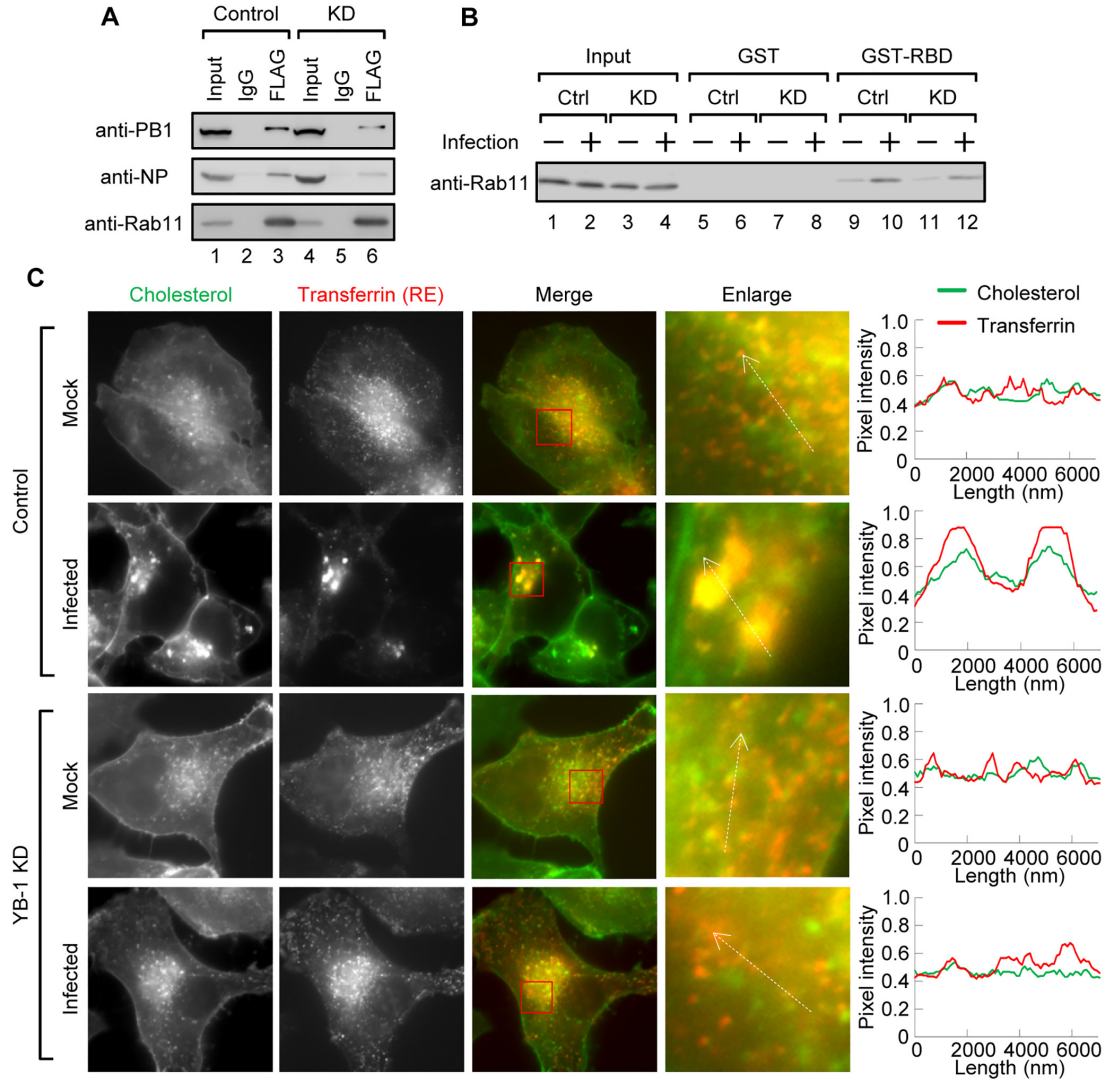
The pericentrosomal ERC is important for the enrichment of vRNP and cholesterol. (A and B) Association of vRNP with Rab11a in YB-1 KD cells. At 48 h post transfection with either non-targeting or YB-1 siRNA, HeLa cells constitutively expressing FLAG-Rab11a were infected with influenza virus at MOI of 10. At 8 h post infection, cells were subjected to immunoprecipitation assays with either non-specific IgG or anti-FLAG antibody (panel A) and GST pull-down assays using GST-RBD (panel B) as descried in Fig 2, respectively. Co-precipitated proteins were analyzed by western blotting with anti-PB1, anti-NP, and anti-Rab11a antibodies. (C) Accumulation of cholesterol in ERC in infected cells. At 48 h post transfection with either non-targeting or YB-1 siRNA, HeLa cells were infected with influenza virus at MOI of 10. At 6 h post infection, cells were pulse-labeled with 100 μg/ml of transferrin conjugated with Alexa 568 (red) for 30 min at 37°C, followed by incubation without Alexa 568-labeled transferrin for 30 min. After fixing in 4% PFA, cells were incubated with 200 μg/ml filipin to visualize cholesterol (green). In panel C, quantitative determination of the each signal (cholesterol, green; transferrin, red) was performed along the white dashed arrows shown within the panel C, enlarged panels. Y-axis indicates the normalized pixel intensity.

ERC is reported to be involved in intracellular sorting and polarized trafficking of apical plasma membrane components [26]. However, details regarding the roles of ERC remain to be clarified. Therefore, we next examined the loading of vRNP onto the recycling endosomes by using YB-1 siRNA to disrupt ERC formation. Cells constitutively expressing FLAG-Rab11a were subjected to immunoprecipitation assays with anti-FLAG antibody (Fig 6A). We found that the amount of PB1 subunit of viral polymerase and NP coimmunoprecipitated with FLAG-Rab11a from YB-1 KD lysates were decreased to approximately 30% of those from control lysates (Fig 6A, lane 6). This result is supported by the fact that vRNP hardly colocalized with Rab11a in YB-1 KD cells as shown in the enlarged panel of Fig 3B. Furthermore, we examined the activation of Rab11a in YB-1 KD cells by GST pull-down assays using GST-RBD. The amount of Rab11-GTP was not changed between the control and YB-1 KD cells (Fig 6B), suggesting that YB-1 KD does not influence the amount of active recycling endosomes. Thus, it is likely that the formation of pericentrosomal ERC is important to load vRNP onto the endosomal vesicles.

Cholesterol is not uniformly distributed in the membrane, and 80–90% of total cellular cholesterol is enriched in the plasma membrane [30]. Although recycling endosomes contain considerably less cholesterol than the plasma membrane, it is known that the endocytic transport pathway through recycling endosomes is important for cholesterol trafficking and homeostasis in cells [31,32]. Therefore, we hypothesized that vRNP is transported to the plasma membrane via recycling endosomes with cholesterol. To test this hypothesis, we observed the intracellular localization of cholesterol in infected cells using the fluorescent cholesterol-binding polyene antibiotic, filipin. Some recycling endosomes were partially colocalized with cholesterol in uninfected cells (Fig 6C). However, along with the formation of pericentrosomal ERC by infection, we found that cholesterol is highly enriched in ERC in an YB-1-dependent manner. Similar results were obtained in A549 cells infected with A/Panama/2007/99, which is one of the representative strains of seasonal influenza A virus (H3N2) (S2 Fig). These findings suggest that vRNP is transported to the plasma membrane via recycling endosome vesicles that contain a higher concentration of cholesterol.

### ERC is involved in viral budozone formation at the plasma membrane

**Fig 7:**
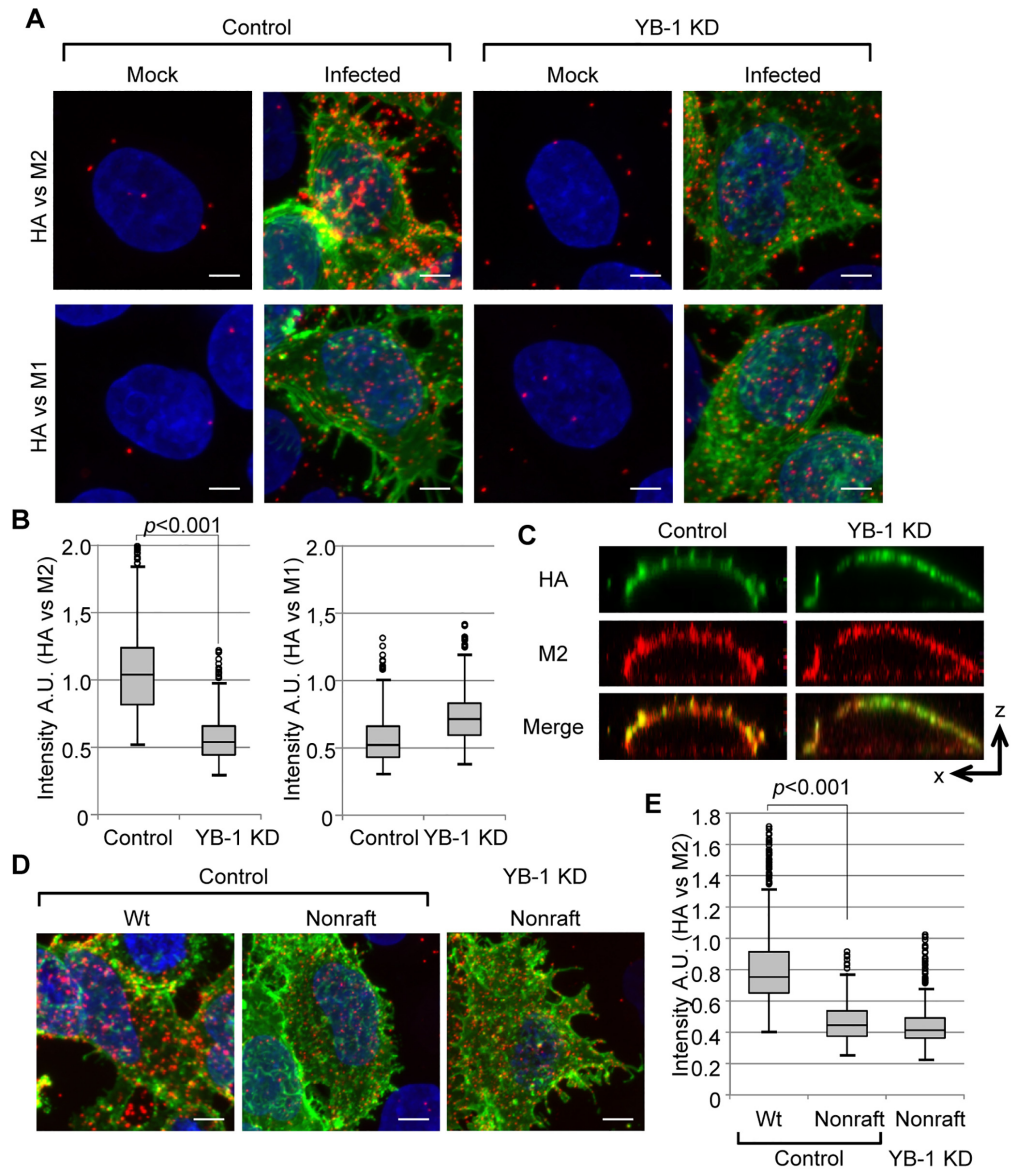
Clustering of HA and M2 requires ERC formation mediated by YB-1. (A, B, and C) *in situ* PLA assays using wild-type virus. At 48 h post transfection with either non-targeting or YB-1 siRNA, HeLa cells were infected with wild-type influenza virus at MOI of 10. At 8 h post infection, cells were fixed and subjected to *in situ* PLA assays with anti-HA and either anti-M1 or anti-M2 antibodies without permeabilization in 0.5% Triton X-100 (red). HA and DNA were counter-stained with anti-mouse IgG conjugated with Alexa 488 (green) and DAPI (blue), respectively. In panel B, the mean intensity of each punctate PLA signal obtained from three independent experiments was quantitated using IMARIS software (*n*>5,000). In panel C, cells were subjected to the indirect immunofluorescence assays with anti-HA (green) and anti-M2 antibodies (red). The single optical sections in the x-z plane are taken. (D and E) *in situ* PLA assays using nonraft HA virus. Control and YB-1 KD cells were infected with a mutant virus at MOI of 10, which has alanine substitutions at I533, Y534, and S535 in the transmembrane domain of HA (nonraft), and subjected to *in situ* PLA assays with anti-HA and anti-M2 antibodies (red). HA and DNA were counter-stained with anti-mouse IgG conjugated with Alexa 488 (green) and DAPI (blue), respectively. In panel E, the mean intensity of each punctate PLA signal obtained from three independent experiments was quantitated using IMARIS software (*n*>5,000). The stacking images along the z-axis were obtained by Maximum intensity projection processing of ZEN 2009 software (Carl Zeiss) (panel A and D). The level of significance was determined by Student’s *t* test. Scale bar, 5 μm.

Some viruses, including influenza virus, are known to utilize lipid rafts for budding from the plasma membrane [33]. Viral budozone formation is thought to be dependent on the spatial assembly of eight-segmented vRNP complexes and viral membrane proteins via clustering of lipid rafts. Although it has been reported that reorganization of cortical actin is required for the control of viral budozone formation [25,34,35], the trigger to initiate the coalescence of lipid rafts is unclear. Thus, we examined whether the pericentrosomal ERC is required for budozone formation by using *in situ* proximity ligation assay (PLA) to detect the proximity between M2 and HA. In the *in situ* PLA system, the theoretical maximum distance between two target proteins is around 40 nm to yield amplified signals. At 8 h post infection, cells were subjected to *in situ* PLA using anti-HA and either anti-M2 or anti-M1 antibodies (Fig 7). Strong punctate PLA signals (red) between HA and M2 or between HA and M1 were observed at the plasma membrane in the infected control cells (Fig 7A and 7B). Although HA and M2 were successfully transported to the plasma membrane in YB-1 KD cells (Fig 7C), the intensity of PLA signals between HA and M2 was significantly decreased by YB-1 KD (*P*<0.001; Fig 7B, left panel). In contrast, the signal intensity between HA and M1 was not decreased in YB-1 KD cells (Fig 7B, right panel). This could be due to the direct binding of M1 with the cytoplasmic tail of HA [19]. Next, we examined whether cholesterol is required for the YB-1-dependent viral budozone formation using nonraft HA mutant virus, which has alanine substitutions at I533, Y534, and S535 in the transmembrane domain of HA. It is reported that this mutant HA rarely associates with lipid rafts and that the apical transport is delayed, but not blocked [36]. At 12 h post infection, a significant amount of HA was observed at the plasma membrane in nonraft virus infected cells (Fig 7D, green). However, the intensity of PLA signals between HA and M2 was dramatically reduced in nonraft virus-infected cells compared with that in wild-type infected cells (Fig 7D and 7E). Thus, as expected, it is likely that most of the signals observed in the *in situ* PLA system were mediated by lipid rafts. Furthermore, in contrast to wild type virus (Fig 7B), the PLA signals between nonraft HA and M2 were nearly unaffected by YB-1 KD (Fig 7E, compare lane 2 with lane 3), suggesting that the interaction of HA with cholesterol is important for YB-1-mediated viral budozone formation.

## Discussion

**Fig 8:**
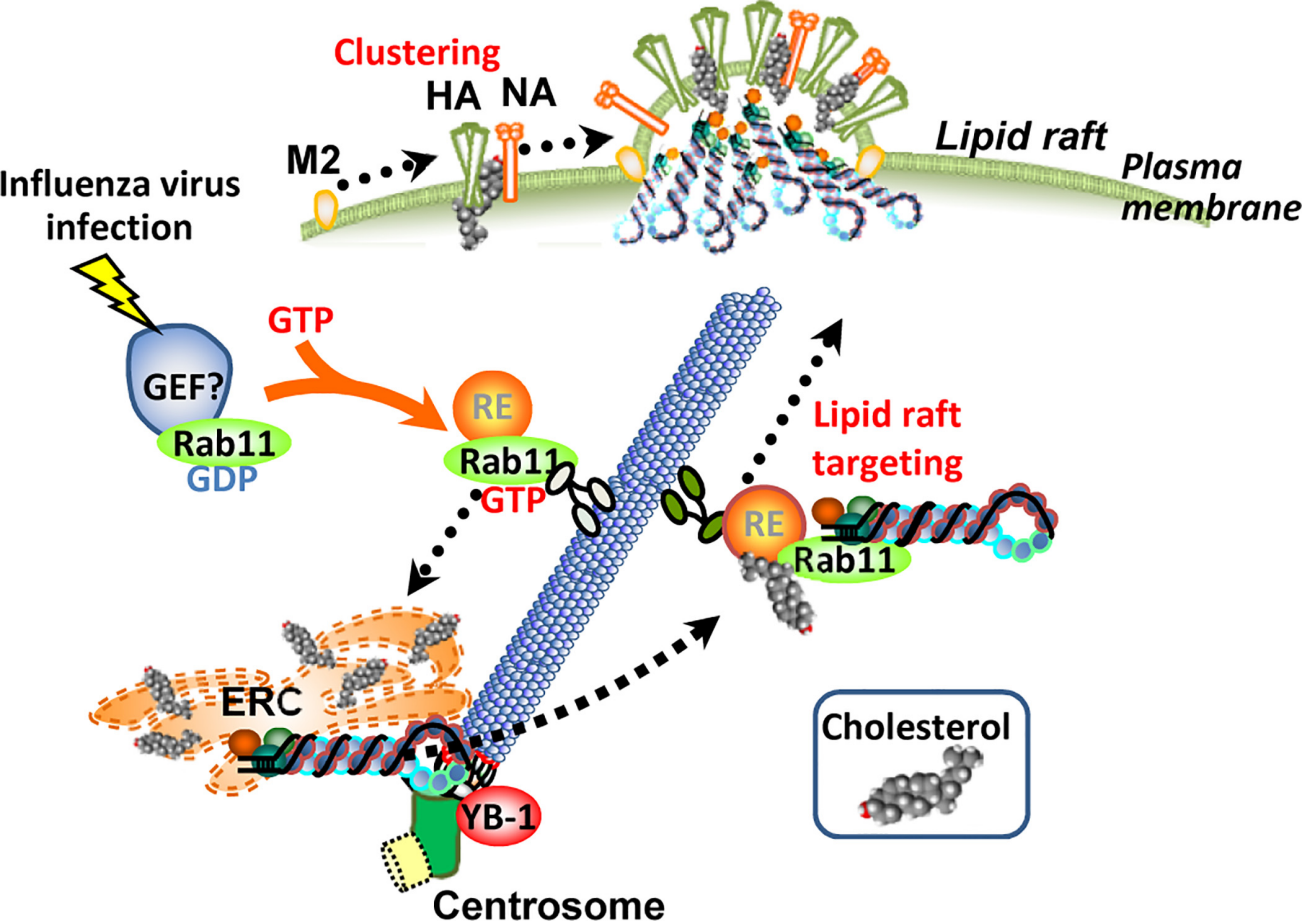
A proposed model. Influenza virus infection induces the accumulation of pericentrosomal ERC through the activation of Rab11a GTPase and microtubule formation from the centrosome. YB-1 is required for anchoring of nucleated microtubules at the centrosome. Along with the formation of pericentrosomal ERC, cholesterol accumulates in ERC with vRNP in an YB-1-dependent manner. The recycling endosome for transport of vRNP emanates from the cholesterol-enriched ERC, and the cholesterol in the recycling endosome may be a trigger for budozone formation concomitantly with vRNP trafficking.

The lipid-lipid, lipid-protein, and protein-protein interactions facilitate the formation of small raft domains into functional platforms for signal transduction, membrane trafficking, and cell adhesion [37–39]. Sphingolipids that have been enriched in these assemblies have saturated and longer acyl chains with larger polar headgroups, so cholesterol functions as spacers between sphingolipids through their acyl chains [40]. This cholesterol-sphingolipids interaction results in the packing and condensing of lipid rafts for their clustering. Fig 7 shows that YB-1 is important for clustering of viral membrane proteins at the plasma membrane through the interaction of viral raft protein with cholesterol. It is noteworthy that the amount of cholesterol at the plasma membrane was unchanged between the control and YB-1 KD cells (S3 Fig), suggesting that small raft domains should be intact at the plasma membrane in YB-1 KD cells. This is possibly due to the fact that the recycling endosomes and TGN contain much less cholesterol than the plasma membrane [41]. However, it is known that moderate changes in the level of cholesterol transported through these compartments appear to have drastic effects on cellular homeostasis [41]. Taking these findings together, we propose that the fusion of cholesterol-enriched recycling endosomes with the plasma membrane induces the accumulation of sphingolipids that contain viral raft proteins which form viral budozone concomitantly with the arrival of vRNP beneath the plasma membrane (Fig 8).

In general, cells acquire cholesterol mainly through receptor-mediated endocytosis of low-density lipoprotein (LDL) [30]. After LDL internalization, LDL-cholesterol is delivered to late endosomes and lysosomes to release the cholesterol molecules from LDL. The majority of cholesterol in late endosomes is then delivered to the plasma membrane. Although the itinerary of cholesterol from late endosomes to the plasma membrane is not clear, it is thought that cholesterol is transported through ER, TGN, and recycling endosomes. We found that influenza virus infection stimulates cholesterol accumulation in ERC (Fig 6C). This could be due to a possibility that the accumulation of recycling endosomes in ERC (Fig 1A) may slow down the delivery of cholesterol to the plasma membrane.

YB-1 is required for centrosome maturation during mitosis [17], but little is known about the function of YB-1 in the centrosome. In infected cells, YB-1 was localized in PCM and formed a radial and toroidal structure around the centriole (Fig 3). It is proposed that the PCM proteins might be assembled based on the nine-fold radial symmetry of the centriole [5,6]. In which case, it is assumed that YB-1 is also a structural component of the PCM matrix for microtubule assembly. It is also reported that YB-1 interacts with microtubules and coats the outer surface of the microtubule wall *in vitro* [42]. Thus, YB-1 may connect microtubules to the PCM matrix by decorating the microtubules’ minus ends.

The spatiotemporal regulation of Rab GTPase activity is of particular importance. Among the several GEFs known to regulate Rab GTPases, no GEF that activates Rab11a has been identified in mammalian cells despite a systematic characterization of the DENN domain subfamily of Rab GEFs [43]. It is necessary to identify the GEFs responsible for virus infection.

Rab11a plays a role in the transport of M2 to the apical membrane [25], although M2 is directly transported through TGN to the plasma membrane [44]. This is due to the fact that Rab11a also functions in constitutive exocytosis from TGN in addition to the recycling processes via ERC [45,46]. In YB-1 KD cells, HA and M2 were successfully transported to the plasma membrane (Fig 7D), suggesting that centrosome maturation by YB-1 is required for their transport through ERC but not through TGN. It has been reported that the minus end of microtubules, which is released from the centrosome, could subsequently be captured by the Golgi membrane and then elongated into linear arrays [47]. Thus, even in the absence of YB-1, the exocytic transport from TGN might be achieved along microtubules that are elongated from Golgi stacks.

The majority of membrane proteins are sorted at TGN before their delivery to the appropriate cell surface domain. In addition to TGN, some other cellular lipid raft proteins, such as TLR4 and EGF receptor, are transported to the plasma membrane through the recycling endosomes [48,49]. Additionally, the transport rates of recycling endosomes are controlled in response to signaling pathways to increase or decrease the surface expression of molecules, such as insulin-regulated glucose transporter GLUT4 [50,51]. In this study, we propose that the recycling endosomes deliver cholesterol to the plasma membrane for not only cholesterol homeostasis, but also lipid raft clustering. Our findings contribute to the understanding of the molecular mechanism of lipid raft clustering in response to several signals that utilize lipid rafts as a platform.

## Materials and Methods

### Biological materials

Influenza virus A/Puerto Rico/8/34 strain and rabbit polyclonal antibodies against PB1, NP, M1, and YB-1 were prepared as previously described [14]. Mouse antibodies against HA (TaKaRa; C179), Rab11a (BD; 47/Rab11), Pericentrin (Abcam; ab28144), α-tubulin (Sigma; DM1A), and a rabbit antibody against M2 (Abcam; ab56086) were purchased. HeLa cells (a gift from Dr. Masa-atsu Yamada of University of Tokyo) were grown in minimal essential medium (MEM) containing 10% fetal bovine serum. Plasmids expressing GFP-centrin-2 and EB1-GFP were prepared as previously described [14]. To establish HeLa cell lines constitutively expressing either GFP-centrin-2 or EB1-GFP, cells were transfected with pSV2-Neo and either pCAGGS-GFP-centrin-2 or pCAGGS-EB1-GFP. The transfected cells were cultured in the presence of 1 mg/ml of G418 for 2 weeks, and then the G418-resistant colonies were isolated. For the construction of plasmid expressing GST-Rab-binding domain (RBD) of FIP2, cDNA was amplified from pCAGGS-FIP2 (provided by Dr. F. Momose, Kitasato University) with primers 5ʹ-CCGGAATTCGAGCTGGTGAAACAC-3ʹ and 5ʹ-ACGCGTCGACTCACGGCACTCTGAG-3ʹ. The cDNA was cloned into pGEX-6P-1. Nonraft HA virus was generously provided by Drs. Y. Morikawa and F. Momose (Kitasato University) [36] and amplified using MDCK cells constitutively expressing HA (provided by Dr. N. Takizawa, Institute of Microbial Chemistry).

### Transferrin uptake

Transferrin conjugated with Alexa 568 was purchased (Life Technologies). Cells were incubated with 100 μg/ml of Transferrin for 30 min at 37°C. After washing with medium, cells were further incubated for 30 min at 37°C and then fixed in 4% paraformaldehyde (PFA).

### Cellular localization of viral RNAs and proteins

Indirect immunofluorescence assays and fluorescence *in situ* hybridization (FISH) assays were carried out as previously described [14]. Briefly, cells infected with influenza virus at multiplicity of infection (MOI) of 10 were fixed with 1% PFA for 10 min and then pre-permeabilized on ice with 0.01% digitonin in PBS for 5 min on ice. After being washed with PBS, cells were fixed in 4% PFA for 10 min and permeabilized on ice with 0.5% Triton X-100 in PBS for 5 min. After incubation in PBS containing 1% bovine serum albumin for 1 h, coverslips were incubated with each antibody for 1 h and then with Alexa Fluor 488-, 568-, and 633-conjugated secondary antibodies, respectively (Life Technologies). After indirect immunofluorescence assays, FISH assays were performed using an RNA probe complementary to the segment 1 virus genome. Images were acquired using confocal laser scanning microscopy (LSM700; Carl Zeiss) or super-resolution microscopy (3D-SIM ELYRA; Carl Zeiss).

### Cholesterol staining

Cells were fixed in 4% PFA for 10 min and then incubated with 200 μg/ml of filipin (Sigma). After washing with PBS, images were acquired by Axio Observer Z1 microscope using 63x Apochromat objective (NA = 1.4) with AxioCam MRm camera (Carl Zeiss).

### Live-cell imaging

Observations were made with Axio Observer Z1 microscope using 63x Apochromat objective. Images were acquired at 1.56-sec intervals for 1 min with confocal laser scanning microscopy (LSM700; Carl Zeiss). All experiments were carried out at 37°C under 5% CO_2_ in a temperature-controlled stage (Carl Zeiss). Sequential images were processed using Image J digital image processing software (National Institutes of Health, Bethesda). The average velocity of the punctate fluorescent signals of EB1-GFP was measured using a manual object tracking plugin, MTrackJ, for Image J.

### *In situ* Proximity Ligation Assay (PLA)

Cells were fixed with 4% PFA, followed by blocking with 1% milk for 30 min. The cells were incubated with mouse anti-HA antibody for 1 h and fixed again in 4% PFA. Cells were then permeabilized with 0.5% Triton X-100 for 5 min and incubated with either rabbit anti-M1 or anti-M2 antibody for 1 h. PLA was carried out using Duolink In Situ PLA kit (Olink Bioscience) according to the manufacturer’s protocol. The mean intensity of the PLA signals was measured using IMARIS software (Carl Zeiss).

### Gene silencing mediated by siRNA

Knockdown of YB-1 was examined as previously described [14]. Briefly, cells (5 x10^5^) were transfected with 30 pmol of siRNA using Lipofectamine RNAi Max (Life Technologies) according to the manufacturer’s protocol.

## Supporting Information

S1 Fig The amount of YB-1 protein in siRNA-treated cells. HeLa cells were transfected with either non-targeting (control; lanes 1–3) or YB-1 siRNA (YB-1 KD; lanes 4–6). After 48 h post transfection, the cells were lysed, and the lysate (5 x10^3^, 1 x10^4^, and 2 x10^4^ cells) were analyzed by SDS-PAGE followed by western blotting assays with anti-YB-1 and anti-α-tubulin antibodies, respectively.
(TIF)

S2 Fig Accumulation of cholesterol in ERC in infected A549 cells. A549 cells were infected with either A/Puerto Rico/8/34 or A/Panama/2007/99. At 6 h post infection, A549 cells were pulse-labeled with 100 μg/ml of transferrin conjugated with Alexa 568 (red) for 30 min at 37°C, followed by incubation without Alexa 568-labeled transferrin for 30 min. After fixing in 4% PFA, cells were incubated with 200 μg/ml filipin to visualize cholesterol (green).
(TIF)

S3 Fig Quantitation of the amount of cholesterol in the plasma membrane. At 48 h post transfection of either non-specific or YB-1 siRNA, cells were collected and swollen in a buffer containing 20 mM Tris-Cl (pH 7.9), 10 mM KCl, and 5 mM MgCl_2_ for 10 min. After passing through a 27-gauge needle, unbroken cells and nuclei were removed by centrifugation at 1,000 xg for 5 min. The supernatant faction was mixed with 72.5% (w/w) sucrose in a buffer containing 10 mM Tris-Cl (pH 7.9), 25 mM KCl, and 5 mM MgCl_2_ to adjust the sucrose concentration to 62.5% (w/w). The sample was transferred to ultracentrifuge tubes, and 55% (w/w) and 5% (w/w) of sucrose buffer were subsequently added, respectively. After ultracentrifugation with SW55Ti at 40,000 rpm for 18 h at 4°C, the plasma membrane fraction recovered between 5% and 55% sucrose layers was collected. The amounts of cholesterol and phospholipids were determined using Amplex Red (Life Technologies) and Labassay phospholipid (Wako) according to the manufacturer’s protocol, respectively. The amount of phospholipids was used as an internal control.
(TIF)

S1 Video Live-cell imaging of EB1-GFP in uninfected control cells, related to Fig 4A. Uninfected cells were subjected to live-cell imaging of EB1-GFP nucleated from the centrosome. EB1-GFP continually emerged from the centrosome. The images were acquired at 1.56-sec intervals for 1 min.
(AVI)

S2 Video Live-cell imaging of EB1-GFP in infected control cells, related to Fig 4A. At 8 h post infection, infected cells were subjected to live-cell imaging of EB1-GFP nucleated from the centrosome. In response to the infection, the nucleation of EB1-GFP from the centrosome was stimulated. The images were acquired at 1.56-sec intervals for 1 min.
(AVI)

S3 Video Live-cell imaging of EB1-GFP in uninfected YB-1 KD cells, related to Fig 4A. Uninfected YB-1 KD cells were subjected to live-cell imaging of EB1-GFP nucleated from the centrosome. The growth rates of EB1-GFP were diversified as shown in Fig 4B. The images were acquired at 1.56-sec intervals for 1 min.
(AVI)

S4 Video Live-cell imaging of EB1-GFP in infected YB-1 KD cells, related to Fig 4A. At 8 h post infection, infected YB-1 KD cells were subjected to live-cell imaging of EB1-GFP nucleated from the centrosome. EB1-GFP moved in a Brownian-like motion in response to the infection. The images were acquired at 1.56-sec intervals for 1 min.
(AVI)
