# Supplementary figures and images for: Influenza Virus Induces Cholesterol-Enriched Endocytic Recycling Compartments for Budozone Formation via Cell Cycle-Independent Centrosome Maturation
Source: PLoS Pathog. 2015 Nov 17;11(11):e1005284. doi: 10.1371/journal.ppat.1005284 (PMC4648529; doi:10.1371/journal.ppat.1005284)

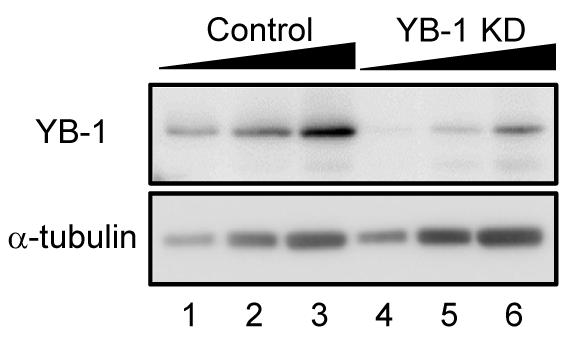

Supplement: S1 Fig — HeLa cells were transfected with either non-targeting (control; lanes 1–3) or YB-1 siRNA (YB-1 KD; lanes 4–6). After 48 h post transfection, the cells were lysed, and the lysate (5 x10^3^, 1 x10^4^, and 2 x10^4^ cells) were analyzed by SDS-PAGE followed by western blotting assays with anti-YB-1 and anti-α-tubulin antibodies, respectively. (TIF) [file ppat.1005284.s001.tif]

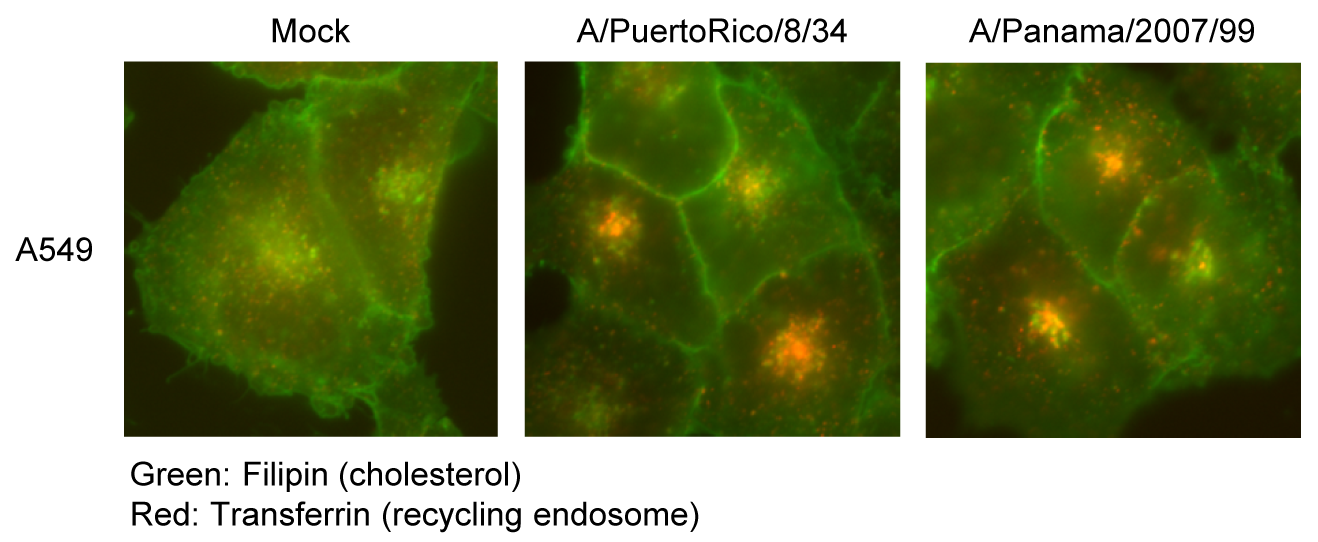

Supplement: S2 Fig — A549 cells were infected with either A/Puerto Rico/8/34 or A/Panama/2007/99. At 6 h post infection, A549 cells were pulse-labeled with 100 μg/ml of transferrin conjugated with Alexa 568 (red) for 30 min at 37°C, followed by incubation without Alexa 568-labeled transferrin for 30 min. After fixing in 4% PFA, cells were incubated with 200 μg/ml filipin to visualize cholesterol (green). (TIF) [file ppat.1005284.s002.tif]

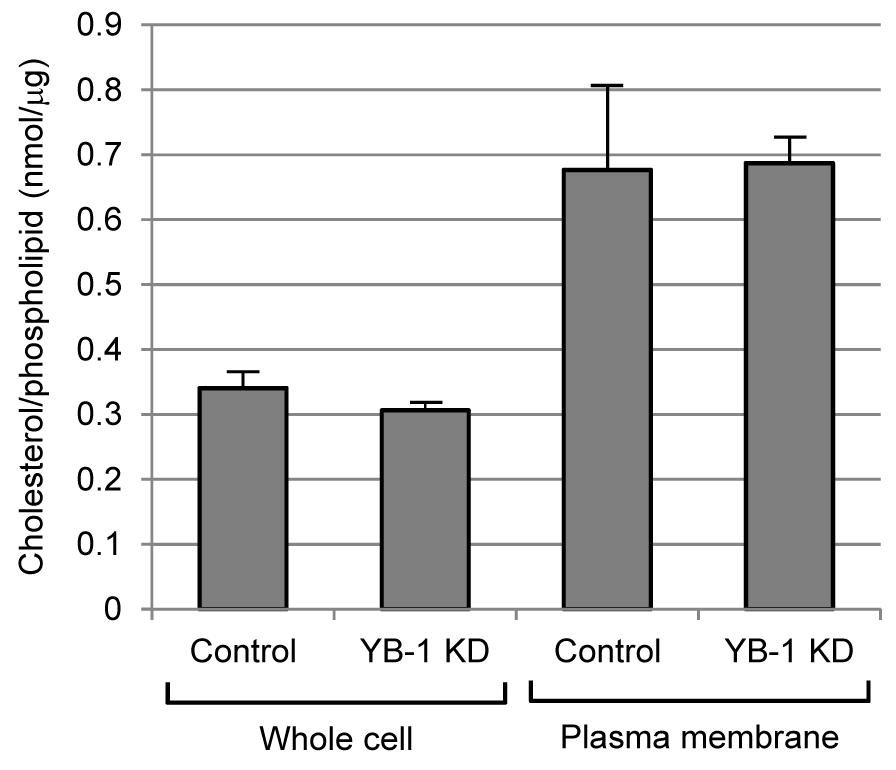

Supplement: S3 Fig — At 48 h post transfection of either non-specific or YB-1 siRNA, cells were collected and swollen in a buffer containing 20 mM Tris-Cl (pH 7.9), 10 mM KCl, and 5 mM MgCl2 for 10 min. After passing through a 27-gauge needle, unbroken cells and nuclei were removed by centrifugation at 1,000 xg for 5 min. The supernatant faction was mixed with 72.5% (w/w) sucrose in a buffer containing 10 mM Tris-Cl (pH 7.9), 25 mM KCl, and 5 mM MgCl2 to adjust the sucrose concentration to 62.5% (w/w). The sample was transferred to ultracentrifuge tubes, and 55% (w/w) and 5% (w/w) of sucrose buffer were subsequently added, respectively. After ultracentrifugation with SW55Ti at 40,000 rpm for 18 h at 4°C, the plasma membrane fraction recovered between 5% and 55% sucrose layers was collected. The amounts of cholesterol and phospholipids were determined using Amplex Red (Life Technologies) and Labassay phospholipid (Wako) according to the manufacturer’s protocol, respectively. The amount of phospholipids was used as an internal control. (TIF) [file ppat.1005284.s003.tif]
